# Safe-by-Design: from Safety to Responsibility

**DOI:** 10.1007/s11569-017-0301-x

**Published:** 2017-08-22

**Authors:** Ibo van de Poel, Zoë Robaey

**Affiliations:** 1 Department of Values, Technology & Innovation, Faculty of Technology, Policy & Management, Delft University of Technology, Jaffalaan 5, Delft, 2628 BX Netherlands; 2 Department of Biotechnology and Society, Faculty of Applied Sciences, Delft University of Technology, Van der Maasweg 9, Delft, 2629 HZ Netherlands

**Keywords:** Safe-by-design, SbD, Nanotechnology, Synthetic biology, Safety engineering, Safety, Design, Uncertainty, Risk, Indeterminacy, Responsibility, Ethics, Users

## Abstract

Safe-by-design (SbD) aims at addressing safety issues already during the R&D and design phases of new technologies. SbD has increasingly become popular in the last few years for addressing the risks of emerging technologies like nanotechnology and synthetic biology. We ask to what extent SbD approaches can deal with uncertainty, in particular with indeterminacy, i.e., the fact that the actual safety of a technology depends on the behavior of actors in the value chain like users and operators. We argue that while indeterminacy may be approached by designing out users as much as possible in attaining safety, this is often not a good strategy. It will not only make it more difficult to deal with unexpected risks; it also misses out on the resources that users (and others) can bring for achieving safety, and it is undemocratic. We argue that rather than directly designing for safety, it is better to design for the responsibility for safety, i.e., designers should think where the responsibility for safety is best situated and design technologies accordingly. We propose some heuristics that can be used in deciding how to share and distribute responsibility for safety through design.

## Introduction

Technologies like nanotechnology and synthetic biology raise new safety issues. This has led to proposals to address safety in both the R&D and design phases of these technologies, which has spurred the concept *safe-by-design* (SbD).

Designing for safety has a long tradition in several engineering disciplines. It has even resulted in new subdisciplines such as safety science and safety engineering. These have proposed various methods to assess risks and to reduce or minimize them through design. Some of these approaches are also directly applicable to emerging technologies but others are not or at least not directly applicable.

In the nanotechnological field, SbD is now an important concept in the European projects Nanoreg2 and Prosafe (http://www.nanoreg2.eu/ and http://www.h2020-prosafe.eu/). More concretely, Jacobs et al. [1] discuss how 12 principles of green chemistry can be abstracted to four more general principles that can be used to design nanotechnological products for safety and sustainability. Also, Morose [2] discusses five design principles for safer nanotechnology.

A recent paper by Hjorth et al. reflects on what the development of SbD for engineered nanomaterials (ENMs) can learn from drug discovery and development (DDD) [3]. SbD can help to address safety already in the R&D and design phases instead of doing toxicity assessments only after ENMs have entered the market. In this connection, the authors refer to the doctrine of “fail early, fail often,” which means doing many safety tests in vitro and in silico to learn about issues and interactions that could decrease safety. However, the authors also point out that the experience with DDD (578 drugs withdrawn from the market for safety reasons) shows that such an approach cannot guarantee absolute safety by design.

While issues of toxicity are paramount to pharmacology and to nanomaterials, synthetic biology faces other kinds of issues as well. Here, one of the biggest problems is the deliberate or unintended release of a modified organism into the environment. In synthetic biology, the concept of SbD has become important in the recent years, in particular through the use of technical safeguards [4, 6, 7 5: 39 ]. One of the most popular technical safeguards found in products using synthetic biology is kill switches. Kill switches allow “switching off” of a cell, so that it stops functioning. However, with the increasing importance of designing for safety, there has also been an increasing realization that safety cannot be achieved through technical means only [5, 8, 9]. There are many aspects at stake that can be called issues of human practices, responsible research and innovation, or cultures of safety. This comes with the realization that safety considered as the absence of risks might not be an achievable goal when dealing with new technologies and their uncertainties.

The concept of SbD can be understood in at least three different ways. One, it may be understood as an approach to risk analysis or risk assessment, implying that risks are already assessed in the design phase. Two, it may be understood as a specific risk management strategy, i.e., addressing safety by design measures, or by built-in safety. An example is kill switches in synthetic biology. Third, SbD might be understood as a result of the design process implying absolute safety and the absence of risk when the technology is implemented.

In this paper, we will employ the second meaning of SbD. We think SbD is particularly interesting as a new way to think of risk management. This second meaning supposes the first, i.e., the assessing of risks during the design phase, but it by no means assumes the third, absolute safety. In fact, absolute safety is impossible and no design strategy can eliminate all risks particularly because of the many uncertainties involved.

We start therefore with pointing out how uncertainty affects the possibilities for SbD. After doing so, we focus on a specific kind of uncertainty, i.e., indeterminacy. With indeterminacy, we refer to the openness of causal chains of human action which may be a source of risks as we will see but also a source of safety. Our main question is how we can best deal with indeterminacy in designing for safety. We argue that in many situations it is better to accept indeterminacy and to use it as a potential source for safety rather than to design it out in an attempt to achieve full safety.

## Uncertainty

SbD approaches aim at addressing risks of new technologies like nanotechnology and synthetic biology already in the design phase to eliminate or at least reduce these risks. Doing so requires anticipating the risks of such technologies. However, anticipating risks is not easy given the uncertainty during the design phase [10]. For new technologies with which there is little operating experience—such as nanotechnology and synthetic biology—this uncertainty is even higher and some risks may only become apparent once the technologies are employed [11].

In this section, we distinguish more specific types of uncertainty and briefly discuss whether SbD can address them, and if so, how. We start here to give the reader a broader picture on how SbD can, or cannot, deal with uncertainty to put our argument in context. Some types of uncertainty we discuss in this section are relevant for our argument below where we focus on exploring one specific type of uncertainty: indeterminacy.

Several classifications of uncertainty have been proposed in the literature, e.g., [12–15]. Building on these, we propose here a classification that is relevant for the current discussion:

1. Risk^1^: We speak of risks if we know what might go wrong (and what the consequences are) and we know the probability of those consequences occurring. Risk is then usually defined as consequences times their probability.^2^
2. Scenario uncertainty: We will speak of scenario uncertainty when we know what might go wrong but cannot meaningfully attach a probability to the occurrence of these consequences. For the specific case of SbD, in which we are interested here, we will understand scenario uncertainty as the case in which we do not know all scenarios (or failure mechanisms) that may lead to an undesirable outcome.
3. Ignorance: We speak of ignorance if we do not know what might go wrong. More specifically, we will understand ignorance for SbD as the situation in which we not only lack knowledge of all failure mechanisms but also do not know certain undesirable consequences that might occur.
4. Indeterminacy: We understand indeterminacy as the situation in which causal chains to the future are still open so that it is indeterminate what will happen. We are specifically interested in cases where indeterminacy is due to the fact that the actors in the value chain, such as users and operators, may employ a technology differently than foreseen or expected by the designers.
5. Normative ambiguity: We understand normative ambiguity as uncertainty or disagreement about values and norms. In contrast to other kinds of uncertainty, this uncertainty is normative rather than descriptive.

To what extent can SbD address these kinds of uncertainty? Risk is the typical starting point of SbD. If we know the risks of a technology, we can look for ways either to eliminate or to reduce the risks. Elimination might be possible by taking away the (root) causes of a risk. So-called inherent safety approaches, for example, try to eliminate hazardous substances, configurations, reactions, or mechanisms and to replace them by others with no or fewer risks. Risk reduction may in principle try to either reduce the likelihood of undesirable scenarios (or failure mechanisms) or reduce the consequences of undesirable scenarios, for example by providing containment.

Not all strategies to eliminate or address risk also examine scenario uncertainty because strategies to reduce risks usually consider known scenarios or failure mechanisms and may therefore overlook others. It is even conceivable that some strategies unintentionally increase the likelihood of certain unknown but undesirable scenarios. Still, several strategies have been developed in safety engineering to address scenario uncertainties. First, the application of safety factors: this means that a construction is made a number of times safer (the so-called safety factor) than the expected or maximum load. Safety factors address not only risks but also scenario uncertainties [17]. It is not immediately clear to us how safety factors would translate to the realm of nanotechnology and synthetic biology. A second strategy is the creation of negative feedback loops in case something goes wrong. An example in traditional engineering is the dead man’s handle that stops the train when the driver falls asleep or loses consciousness. An example from biotechnology and synthetic biology are genetically modified mechanisms that are cripple so they cannot survive outside of the laboratory. Might they escape, they most likely will die. A third strategy is known as “multiple independent safety barriers.” This means that safety does not rely on one safety barrier which might fail but on several barriers that, preferably, cannot be subject to the same failure mechanisms. One possible safety barrier is containment of a dangerous substance in the event of a leak.

A fourth strategy is inherent safety. In safety engineering, inherent safety refers to the elimination of hazards, for example, by replacing dangerous substances or processes by less dangerous ones [16]. Also for nuclear energy reactors, inherent safety is understood as the elimination or exclusion of inherent hazards [18]. However, in synthetic biology, a somewhat different meaning of inherent safety has come in vogue, namely built-in safety. Inherent safety here refers to building safety locks or biocontainment into new organisms [6: 40]. An example is having an engineered organism that can only function on a specific substrate thus reducing the risk of it spreading to undesired destinations. Genetic safeguards, like auxotrophy and kill switches, are currently already used for genetically modified micro-organisms. Synthetic biology may contribute to improved safety locks with higher safety levels [7]. However, such safety measures cannot eliminate all safety hazards [6: 40]. In fact, genetic safeguards or safety locks do not *eliminate* hazards but rather reduce them by building in negative feedbacks loops or additional containment layers. It is estimated that still one cell in a million escapes such engineered safety mechanisms [6: 43]. By combining different safety locks, this factor may be further reduced.

There are several strategies to address scenario uncertainties in addition to risks. However, one must realize that addressing only known hazards may inadvertently impact the ability for SbD to approach all uncertainties [19]. This brings us to the topic of ignorance.

Ignorance is obviously more difficult than scenario uncertainty to include in design because we do not know what can go wrong and therefore cannot design precautions. Sometimes, unknown hazards may be addressed by some of the approaches from safety engineering discussed above. An example is kill switches in synthetic biology. They cannot only help against unknown failure scenarios but also against consequences that are not foreseen or unexpected because they help prevent modified organisms from spreading beyond their intended environment.

However, the functioning of kill switches—and other forms of built-in safety such as safety locks—is still vulnerable to ignorance. One vulnerability is the possibility of genetic mutation, so that kill switches no longer work or are less effective. If it is recognized that there is ignorance about, for example, the possibility of mutation, we speak of recognized ignorance that can, in part, be explored in risk assessment and in design. However, ignorance may also be (initially) unrecognized. In such cases, we do not know that we do not know certain things (so-called unknown unknowns). A famous example is asbestos that proved to have extremely harmful health effects causing diseases including asbestosis and mesothelioma [20]. A more recent and innocent example is titanium dioxide nanoparticles in sunscreens used by construction workers that damaged prefabricated steel roofs when perspiration drops including the sunscreen fell on the material [21].

Generally speaking, addressing unrecognized ignorance requires approaches that extend beyond the design phase as some risks only become clear during employment of a technology in society. One possible approach is adaptive risk management [22, 23]. Rather than relying on full anticipation or prediction of risks, adaptive risk management relies on organizing a learning process, both with respect to what the risks are and how to best manage them. Adaptive risk management also has consequences for design. For example, innovations can be designed to be flexible so that they can be adapted or used differently might new risks emerge. Another strategy is to design applications in ways that their risks can be better monitored or traced back to a specific application if (unexpected) safety problems arise.

Let us now turn to indeterminacy. Indeterminacy refers to the openness of causal chains, in particular due to the unpredictability of human action. One way in which indeterminacy may lead to hazards is through human error [24]. Although it is sometimes thought that decreasing the variability of human action decreases the likelihood of human errors and therefore increases safety, James Reason argues that the human possibility to improvise may be crucial to react to (unexpected) hazards and is therefore also a source of safety [25]. Basically, there are two opposing ways to face indeterminacy. One is to design out indeterminacy as much as possible. The idea behind such an approach is that risks become more predictable and therefore more manageable and containable. Rather than relying on users and other actors, this makes the technology safe-by-design. The other approach might be called embracing indeterminacy. It assumes that indeterminacy is not only a liability but also an asset as it opens the possibility to use the expertise and insights of other actors in the value chain, like those of operators and users, to identify risks unknown during the design phase. Below, we discuss these two approaches and argue for a middle ground that takes the shared responsibility of designers and other actors for safety as a starting point. First, however, it is worthwhile to discuss briefly the fifth type of uncertainty.

Normative ambiguity refers to the fact that values and norms may be uncertain and contested. In the case of SbD, what may be conceived as a risk by one party may be seen as an opportunity by another. For example, think of the possibility to use synthetic biology for terrorism or warfare, for example, by creating a new deadly virus. For terrorist groups, this may be an opportunity rather than a risk. In such cases, one might argue that, from a moral point of view, such uses are clearly unacceptable; thus, there is no real normative (or at least no moral) ambiguity. However, such cases may be characterized by normative ambiguity in another way. Technologies typically serve or need to respect more than one value. Apart from safety, other values, such as sustainability, privacy, and fairness, will be relevant depending on the application. In cases where safety is attained at the cost of another value, this may lead to normative ambiguities as it is unclear how to weigh or balance the different values. This points to a more general limitation of SbD approaches because they forefront one specific value, i.e., safety, while design often requires considering a range of potentially conflicting values.

## How to Deal with Indeterminacy in SbD?

We now come to the main issue we want to discuss: How to incorporate indeterminacy in SbD? First, we present arguments for why it may be a good idea to design out indeterminacy. Second, we discuss two arguments for why it might be better *not* to design out indeterminacy. Third, we integrate both points of view by arguing for an approach that properly attributes the responsibility for safety rather than just focusing on safe-by-design.

### Arguments for Designing Out Indeterminacy and Idiot-Proof Design

SbD is aimed at identifying the risks of a technology during the early phases of technological development (R&D, design) and then eliminating or at least reducing these risks. Risk identification will often result in the identification of several scenarios (or failure modes) that lead to unsafe situations or undesirable outcomes. In at least some of these scenarios, human behavior will have a prominent role, that is to say the realization of at least some failure scenarios will depend on the behavior of actors in the value chain. The likelihood of these undesirable scenarios can be decreased in at least two ways, both of which require the designing out of indeterminacy.

One way is to make the occurrence of undesirable scenarios less sensitive to human behavior. In this approach, the non-human elements are designed so that undesirable scenarios are less likely to occur even if humans behave unexpectedly or unfavorably. For example, an electric saw may be designed to be safe even if the user uses it inappropriately or for goals for which it was not designed. In this approach, human behavior as such does not become less indeterminate, but the design is safe(r) despite human indeterminacy.

Another approach is to steer human behavior. This can be done through organizational provisions such as (company) rules and regulations or through education and training of operators (or users). It can also be done through technical design ranging from ways that, for example, allow users to interact with the artifact only in predefined ways to efforts to persuade or nudge users (and other actors) into certain behavior as in persuasive technology [26, 27].

What these approaches have in common is that they aim at decreasing the consequences of indeterminacy of actors’ behavior. This may be done to increase safety but behavior changing or influencing technologies are also employed to attain other values such as sustainability.

When it comes to safety through design, a notion that has been put forward is that of idiot-proof design. The idea here is to make design safe despite the idiotic things that users (and others) may do. Positively formulated, idiot-proof design may be seen as the designer assuming responsibility for safety and as a way to shield the user, and others, from harm. It also implies that a technology can be designed so that it can only be used in one particular way and that nothing could go “wrong.” However, as `Bucciarelli [10: 49] points out, idiot-proof design reflects a disdain for the user: “By design we insure that our user can only act as an idiot; he or she has no other recourse.” Moreover, idiot-proof design may give the wrong impression that absolute safety is attainable; and, even worse, it may be counterproductive as it decreases the possibilities to recognize unknown hazards, which may well decrease the overall level of safety rather than increase it. Let us therefore now discuss possible arguments against designing out indeterminacy in SbD.

### Two Arguments Against Designing Out Indeterminacy

We will consider two arguments against designing out indeterminacy in SbD. The first argument is that attempts to design out indeterminacy reduce the possibility to address unknown or unexpected risks and therefore may be ineffective or even counterproductive for safety. The second argument is that designing out indeterminacy implies denying, or at least downplaying, the role of actors other than the designers in the process of technological development and deployment and therefore is undemocratic.

Let us start with explication of the first argument. As we have seen, designing out indeterminacy may increase safety regarding known risks because it lowers either the likelihood, or effects, of failure scenarios in which human behavior plays an important role. This strategy may be effective for reducing not only risks but also uncertainty, at least in situations where certain human behavior is likely to be unsafe, even if we do not exactly know the scenarios or failure modes leading to those unsafe situations. However, when it comes to ignorance, or unknown risks, designing out indeterminacy is less likely to be effective and may be counterproductive. By designing out indeterminacy, technologies are unlikely to be adaptive and flexible thus less able to be redesigned to accommodate unknown risks that arise.

As Buciarelli (1985) points out the idea of idiot-proof design might well originate in a culture or ideology of predictability and rationality in design and while it may not be bad to try to predict or to be rational, one should be aware that not everything in design can be predicted. Thus, while designing out indeterminacy may, on occasion, lead to safer products, it may also lead to the misconception that all risks can be foreseen in the design, and—more harmfully—it may reduce adaptability and flexibility and thereby the ability to deal with unexpected hazards. Moreover, as Wynne [28] has pointed out, engineers and scientists often operate from the assumption that technology will behave according to rules, while in practice technology is unruly and use practices will not follow the intentions of designers. The assumptions about user behavior that designers build into their technologies may be unrealistic not because users are malevolent but because the real-world is less predictable and messier than assumed. These unrealistic assumptions in turn may become a source of new hazards rather than increased safety as intended.

Our point is not only that designing out indeterminacy, or idiot-proof design, will result in designs that are less flexible and adaptable but also that it denies actors other than the designers a role in achieving safety and therefore denies the potential contribution of these actors to safety. Users, and other actors such as operators, may have expertise and skills, lacking in the designers, that can improve safety. First, as the actual employers of the technology, they will often be among the first to experience or note unexpected hazards or unintended consequences. Reacting to unexpected hazards often requires the ability to improvise. Improvisation requires one to embrace the indeterminacy of human action. Moreover, through appropriation, they will make the technology their own and they will develop expertise and skills to employ the technology safely, often in other ways than foreseen by the designer. This means that they cannot only be a source of danger but also be a source of safety. In some cases, it may be the users rather than the designers that are the experts in how to employ a technology safely. They can, however, only play a role as experts if they are not treated as idiots.

Taking users seriously is critical when considering the promises of applications in nanotechnology and synthetic biology because products containing these applications are likely to be distributed among many actors. For instance, sunscreen containing nanoparticles, or a biosensor in food packaging might become a normal product in every household. As we have learned to deal safely with battery disposal, or super glue use, we should also learn to treat with these new applications safely instead of taking their safety for granted.

We now turn to the second argument against designing out indeterminacy: it is undemocratic. The idea here is that technology plays an important role in shaping our modern society and our lives. Winner [29] for example compares technology with laws that are subject to democratic decision-making. Sclove [30] maintains that technologies function in ways similar to social structures and argues for the design of democratic technologies. If technologies indeed shape our lives and society, it is desirable that users and other stakeholders also have a say in how they are designed. This has led several authors to argue for more participatory forms of design and technological development [31].

By designing out indeterminacy designers deny users, and other stakeholders, possibilities to shape a technology during its use phase. As Buciarelli (1985: 56) notes the effect of idiot-proof design “is to forbid trespassing with the world of design. As such the designer’s intent may appear to be to protect the design itself from tampering as much as to shield the user from harm.” So conceived, designing out indeterminacy, and in this way denying users and other stakeholders, a role in shaping (the use of) technology is not politically innocent, but is a way to sustain the hegemony of the designers (and the organizations for which they work) in the shaping of technology.

Looking at synthetic biology specifically, but perhaps also nanotechnology in the future, open laboratories and the DIY movement present a case for concern but also opportunities. Proponents of this movement want to make a number of applications, such as pharmaceuticals, more accessible to citizens. For example, the Counter Culture Labs in Oakland claim, “Biology is the technology of the 21st century, and has the potential to affect our lives as much as or more than computers did in the 20th century. Our goal is to demystify and democratize this technology, putting tools into the hands of those who want to learn. We believe in the power of diversity and peer-to-peer education; everybody has something to teach and everybody has something to learn. Whether you’re ready to start testing that cutting-edge, pre-startup research idea, or just want to play with some bioluminescent algae, this is the place for you!”.^3^ At the same time, these citizen initiatives cause biosafety and biosecurity experts concern. Nevertheless, these laboratories typically have to meet the same requirements as regular laboratories in terms of permissions to carry out certain types of activity. There have been no recorded breakthroughs in these laboratories yet, but their ambition is there. Because this movement seems to be on the rise, it is important not to dismiss it.

## Design for Responsibility

We will now rephrase the discussion above in terms of responsibility to identify a middle ground between designing out indeterminacy and embracing it in a way that does justice to the arguments made above. A first thing to note is that designing out indeterminacy may be a way for designers to assume responsibility for the safety of their design and to protect others from harm. Taking this responsibility is laudable and positive. What nevertheless is problematic is that, by designing out indeterminacy, they assume responsibility in a way that denies responsibility (for safety) to other actors. The solution to this problem should not be sought in giving all responsibility for safety to the users, or other stakeholders and actors in the value chain, but rather in a model of shared responsibility for safety. Such shared responsibility may be more effective in achieving safety, as it can also tap into the resources of users and may be more fair, as it also provides opportunities to actors other than the designers to shape technology. If one accepts this argument and aims at shared responsibility for safety, the next question that arises is how responsibility should be shared and distributed over the various actors involved. Another question is whether we can design for responsibility (for safety) and if so how.

Let us start with how to share and distribute responsibility over the actors involved. Fahlquist et al. [32: 486] suggest several heuristics for sharing responsibility in design. A responsibility distribution should be complete, fair, and effective. Complete means that for each relevant (safety) issue at least one actor is responsible. Fair means that responsibility should be distributed in a way that it is perceived as fair by the actors involved. Among others, this implies that actors should only be attributed responsibilities they can live by. In connection with our earlier discussion, it also implies that each agent should have a fair amount of responsibility to be able to help shape the design and employment of a technology. Effective means that responsibility is so distributed that safety issues are dealt with effectively. To these criteria, Fahlquist et al. [32] add a fourth, which they call cultural appropriateness: “Design should strike the balance between individual and collective responsibility in a way that is culturally appropriate.”

It should be noted that the question what responsibility distribution is best in the light of the mentioned criteria will be context-sensitive. For some products, users or operators are likely to have much expertise, and it would be most effective to give them much responsibility for safety. In other cases, this may be different and designing out some indeterminacy may be quite acceptable. A special case is dual use [33], a term that refers to the possibility that technologies that have been developed for civilian purposes can also be used for military purposes. What is interesting for our discussion is that some technologies that have been designed for good purposes may also be intentionally used for bad ones. Synthetic biology is a case in point. It may be used to design organisms with useful properties as well as to design harmful organisms, such as pathological viruses that may be used for terrorist purposes and warfare. Sometimes one and the same technology may be employed for both useful and harmful goals. In such cases, designing out indeterminacy of users may sometimes be a good strategy, and it may sometimes be effective for safety to deny users certain responsibilities. At the same time, we should be aware that we can probably never fully design out indeterminacy. Completely avoiding the possibility of dual use by design seems impossible. Moreover, some dual uses can be difficult to foresee, and addressing them requires adaptability and flexibility. It may be more effective to allow actors involved in use and operation, or regulators, the ability to improvise. There is no exact recipe for how to do this, but it requires careful deliberation about what indeterminacy to accept and what responsibilities to allocate to which actors through design.

We now turn to the question of how we can design for responsibility. Fahlquist et al. [32] provide heuristics for designing for responsibility. We will not discuss them all but rather highlight some. In relation to the issue of designing out, or embracing indeterminacy, their first two design heuristics are important here:

- H1. Moral agency: Design should not diminish the moral agency of users, operators, and other stakeholders. ….
- H2. Voluntariness or freedom: Designs should respect or improve the voluntariness of actions, e.g., by increasing the options of actions for users, operators, and other stakeholders [32: 484].

At the same time, they suggest that it might be acceptable to design for behavior change under certain conditions, which—as we have discussed above—is aimed at decreasing indeterminacy through design. They, however, believe that this is only acceptable if it does not decrease the moral agency of actors and their ability to reflect on their actions. More specifically, they provide the following relevant heuristics:

- H6. Behavior: Design should encourage morally desirable behavior of users. It should, however, do so in a way that respects design heuristic H1, H7, and H8.
- H7. Capacity: Design should encourage the capacity of users, operators, and other stakeholders to assume responsibility as a virtue, i.e., their ability to reflect on their actions and their ability to behave responsibly.
- H8. Virtue: Design should foster virtues in users, operators, and other stakeholders. … [32: 485].

Another way in which one can design for shared responsibility is through the design of use plans which allow for transferring responsibilities to the users of a technology [34, 35]. According to Houkes and Vermaas [36], the design of artifacts always includes the design of use plans. A use plan is a sequence of actions with an artifact that will lead to the realization of a goal. Use plans may be communicated through the manual of a product but also in other ways, and users may develop their own use plans that deviate from the use plans of the designers.

Robaey [35] discusses conditions under which the transfer of ownership of an artifact to users, or other agents, corresponds with a transfer of moral responsibility for appropriate use considering the potential hazards of a technology. Although her focus is on (genetically modified) seeds, several of her conditions are also relevant for our discussion here. First, she postulates that there should be at least be one rational use plan that does not result in unacceptable harm from the use of the technology. This use plan should be executable; it should be communicable and it should be context-sensitive in the sense that it considers the possibilities and skills of the users. The use plan should also be adaptable when and where relevant for preventing harm from the use of the technology. But perhaps most important for our discussion is her fourth condition:

The new owner j has epistemic access to the technology. This means that the technology does not remain a black box for j; instead, owner j should have the possibility to change and manage the technology in a context-sensitive manner [35: 780]

This condition is required so that the (new) user can learn about a technology and its hazards and can adapt the use of it to avoid or reduce hazards. More specifically, Robaey argues that epistemic access to the technology is required for a user to employ her forward-looking responsibility for avoiding or decreasing hazards. Employing this responsibility entails acquiring and cultivating a range of epistemic virtues. Here, by epistemic virtues, we understand virtues that are responsibility conducive such as impartiality, intellectual courage, and community [37]. In other words, epistemic virtues are virtues that allow us to learn in a critical way, also known as doing good science. This learning in a critical way is however not reserved for scientists and can take different shapes for different societal actors [38]. So conceived, epistemic access to a technology is required for the user to assume responsibility and the related virtues. Robaey’s fourth requirement can be seen as a further development of heuristic 7 and 8 from Fahlquist et al. [32] for the case of transferring responsibility to users.

## Conclusions

New applications of nanotechnology and synthetic biology are likely to bring new hazards. Proactively addressing such hazards already during the R&D or design phase as is done in SbD is desirable. SbD is thus useful as a safety design strategy but it should not be understood as an outcome, i.e., it should not be confused with absolute safety, which is unattainable. Also, the association of SbD with inherent safety is somewhat confusing. In synthetic biology, inherent safety has come to refer to built-in safety, which is never completely fail-safe, while originally in safety engineering it referred to the *elimination* of certain inherent hazards. Moreover, if SbD focuses too much on known risk and expected scenarios, and attempts to achieve safety by designing out the user, it is in danger of missing opportunities to make technological applications safer. Designing out indeterminacy decreases, rather than increases, the ability to deal with unexpected or unknown risks; it neglects the skills and capacities of users to help achieve safe use of products; and it is undemocratic. The solution is not to be sought in transferring all responsibility to the users of a technology (or to other stakeholders) but rather in a model of shared responsibility. This requires deliberation about how responsibility for safety is best shared among the various actors involved, considering the completeness, fairness and effectiveness of a responsibility distribution in attaining safety. How this balance is best struck is probably different from product to product and may also depend on the cultural context. We have proposed some requirements and heuristics for designing for the responsibility for safety. It must be admitted that these are still very general and they will need further specification for specific products or classes of products to become effective. Our point is, however, more general: it is better to design for the responsibility for safety than to try to make products safe-by-design by designing out all human indeterminacy thus barring options for creatively and adaptively attaining safety in real-world situations. The result of our discussion is not just that we should replace safety by responsibility for safety but also that we should be aware that in the design of any technology a range of values is at stake, values that on occasion may be in conflict. We should therefore be aware that the attainment of one value may come at the cost of others. There might not be an optimal solution, but we believe that an awareness of both the multiplicity of values and the fundamental uncertainty—which comes in different guises—in design is critical to the design of products that are safe and serve better a wide range of human values.

## Footnotes

1 Situations of risk are typically characterized by statistical uncertainty rather than by scenario uncertainty that we distinguish below.

2 In the literature also, other definitions of risk can be found, see for example [16].

3 Counter cultures lab - About -https://counterculturelabs.org/?page_id=9.
